# Assessment of the causal association between celiac disease and cardiovascular diseases

**DOI:** 10.3389/fcvm.2022.1017209

**Published:** 2022-10-21

**Authors:** Jian Huang

**Affiliations:** Clinical Laboratory Center, The First Affiliated Hospital of Guangxi Medical University, Nanning, China

**Keywords:** celiac disease, cardiovascular disease, mendelian randomization, causal effect, summary statistics

## Abstract

**Background:**

Epidemiological studies have reported inconsistent results of the association between celiac disease (CD) and cardiovascular diseases. Moreover, the causality remains largely unknown. Therefore, we aimed to investigate whether CD is causally associated cardiovascular diseases, including ischemic stroke, large artery stroke, cardioembolic stroke, small vessel stroke, coronary heart disease, myocardial infarction, angina, heart failure, atrial fibrillation, and venous thromboembolism using an mendelian randomization (MR) approach.

**Methods:**

Summary-level data for CD were derived from a large-sample genome-wide association study (GWAS) including 12,041 CD cases and 12,228 controls of European ancestry. The corresponding data for ischemic stroke (34,217 cases and 406,111 controls), large artery stroke (4,373 cases and 406,111 controls), cardioembolic stroke (7,193 cases and 406,111 controls), small vessel stroke (5,386 cases and 192,662 controls), coronary heart disease (22,233 cases and 64,762 controls), myocardial infarction (11,622 cases and 187,840 controls), angina (18,168 cases and 187,840 controls), heart failure (47,309 cases and 930,014 controls), atrial fibrillation (60,620 cases and 970,216 controls), and venous thromboembolism (9,176 cases and 209,616 controls) were obtained from the IEU GWAS database. We calculated the causal effect using the inverse variance weighted method. Sensitivity analyses and leave-one-out analyses were performed to ensure the consistency and robustness of causal estimates.

**Results:**

The MR inverse variance weighted estimates indicated no causal effect of genetically predicted CD on ischemic stroke (OR = 1.001, 95% CI: 0.984-1.018), large artery stroke (OR = 1.003, 95% CI: 0.961-1.048), cardioembolic stroke (OR = 1.009, 95% CI: 0.977-1.042), small vessel stroke (OR = 1.023, 95% CI: 0.981-1.066), coronary heart disease (OR = 0.995, 95% CI: 0.977-1.013), myocardial infarction (OR = 0.994, 95% CI: 0.959-1.030), angina (OR = 1.006, 95% CI: 0.981-1.032), heart failure (OR = 0.999, 95% CI: 0.982-1.016), atrial fibrillation (OR = 1.000, 95% CI: 0.990-1.011), and venous thromboembolism (OR = 1.001, 95% CI: 0.971-1.032). Sensitivity analyses using the MR-Egger, weighted median, and simple mode methods yielded similar results. No evidence of horizontal pleiotropy was identified (MR Pleiotropy Residual Sum and Outlier global test and MR-Egger intercept with *P* > 0.05).

**Conclusion:**

Our findings do not support a causal contribution of CD itself to ischemic stroke, large artery stroke, cardioembolic stroke, small vessel stroke, coronary heart disease, myocardial infarction, angina, heart failure, atrial fibrillation, and venous thromboembolism risk.

## Introduction

Celiac disease (CD) is a highly prevalent chronic intestinal disease induced by dietary gluten in genetically susceptible individuals. CD affects about 1% of the general population in Western countries and its prevalence is increasing (1). CD pathogenesis is related to immune-mediated mechanisms. Although CD primarily affects the gastrointestinal tract, it is universally recognized that CD is a systemic autoimmune condition that may presents with a broad spectrum of symptoms (1). Given that several other systemic autoimmune diseases such as primary Sjögren syndrome and rheumatoid arthritis have been found to be linked with atherosclerosis development and a greater risk of cardiovascular events (2–4), a number of observational studies have attempted to assess the relationship between CD and cardiovascular disease. However, the scientific evidence is still scant and contradictory (5–9); not all studies have revealed a positive association. Data from observational studies are sensitive to a multitude of confounding factors (10, 11); the potential causality of CD on cardiovascular disease can not be established in observational studies.

Mendelian randomization (MR) is a causal inference approach using genetic variants (single nucleotide polymorphisms [SNPs]) as instrumental variables for an exposure of interest to investigate if the exposure is an causal contributor for a health outcome (12). It is widely accepted that MR is less prone to residual confounding and reverse causality owing to the use of randomly allocated instrumental variables. Using large-scale genome-wide association study (GWAS) summary-level data, MR has proven to be an effective and reliable alternative to randomized controlled trials for evaluating causality (12). In the present study, we aimed to investigate the causal effect of CD on cardiovascular diseases, including ischemic stroke, large artery stroke, cardioembolic stroke, small vessel stroke, coronary heart disease, myocardial infarction, angina, heart failure, atrial fibrillation, and venous thromboembolism using an MR approach.

## Materials and methods

We performed two-sample MR using GWAS summary data. No ethical approval was required for our study, because we only used publicly available summary data. For avoiding the influence of population structure, our study was restricted to participants of European ancestry. We reported the MR study following the recommendations of the STROBE-MR Guidelines (Supplementary STROBE-MR Checklist).

### Instrumental variable selection

In this MR study, GWAS summary statistics were obtained from the IEU GWAS database^1^. Developed by the Medical Research Council Integrative Epidemiology Unit at University of Bristol, the IEU GWAS database contains freely available GWAS summary-level data for a variety of human phenotypes. The summary statistics for CD were obtained from a GWAS dataset (GWAS ID: ieu-a-1058) built by Trynka et al. (13), including 12,041 cases and 12,228 controls of European ancestry. CD was defined based on standard clinical criteria, compatible serology, and small intestinal biopsy. More details on the characteristics of the CD cases can be found in the original study (13). We identified single nucleotide polymorphisms (SNPs) strongly associated with CD at the genome-wide significance threshold (*P* < 5 × 10^–8^). For minimizing MR biases caused by correlation between SNPs, instrumental variables were restricted to independent SNPs without linkage disequilibrium (R^2^ <0.001). We did not use palindromic SNPs with intermediate allele frequencies, since they may invert the direction of a causal effect. Instrumental variable selection was done using the R package ‘‘TwoSampleMR’’ version 0.5.6^2^ (14, 15). Fifteen instrumental SNPs were identified for CD. A list of the instrumental variables is shown in Supplementary Table 1.

### Outcome data source

The outcomes analyzed included ischemic stroke, large artery stroke, cardioembolic stroke, small vessel stroke, coronary heart disease, myocardial infarction, angina, heart failure, atrial fibrillation, and venous thromboembolism. Summary-level data for these outcomes were obtained from the IEU GWAS database. Table 1 shows the detailed information on the outcome datasets. If instrumental SNPs were missing in the outcome dataset, we applied proxy SNPs based on a linkage disequilibrium cut-off of R^2^ ≥0.8. We harmonized all instrumental variables for each trait to ensure the genetic associations reflect the same effect allele. All data applied were derived from individuals of European ancestry.

**TABLE 1:** Detailed information on the outcome GWAS datasets.

Outcome	Author or Consortium	Year	PMID	GWAS ID	Population	Cases/controls	Number of SNPs
Ischemic stroke	Malik R	2018	29531354	ebi-a-GCST005843	European	34217/406111	7537579
Ischemic stroke (large artery atherosclerosis)	Malik R	2018	29531354	ebi-a-GCST005840	European	4373/406111	7992739
Ischemic stroke (cardioembolic)	Malik R	2018	29531354	ebi-a-GCST005842	European	7193/406111	7954834
Ischemic stroke (small-vessel)	Malik R	2018	29531354	ebi-a-GCST005841	European	5386/192662	6150261
Coronary heart disease	Schunkert H	2011	21378990	ieu-a-8	European	22233/64762	2420361
Myocardial infarction	FinnGen	2021	NA	finn-b-I9_MI_STRICT	European	11622/187840	16380431
Angina	FinnGen	2021	NA	finn-b-I9_ANGINA	European	18168/187840	16380426
Heart failure	Shah S	2020	31919418	ebi-a-GCST009541	European	47309/930014	7773021
Atrial fibrillation	Nielsen JB	2018	30061737	ebi-a-GCST006414	European	60620/970216	1030836
Venous thromboembolism	FinnGen	2021	NA	finn-b-I9_VTE	European	9176/209616	16380466

SNP, single nucleotide polymorphism; GWAS, genome wide association study.

### Statistical analyses

We measured the strength of the instrumental SNPs by calculating the F statistic as previously described (16); it is widely accepted that an *F*-statistics >10 makes weak instrument bias unlikely (17). We investigated the genetically predicted effects of CD on cardiovascular disease risk using the inverse variance weighted method. This method assumes that all instrumental variables are valid and can provide the highest precision (18, 19). However, it is sensitive to pleiotropy, which describes a situation where a genetic variant influences two or more phenotypes. Thus, we used the Mendelian randomisation Egger Regression (MR-Egger), weighted median, simple mode, and the Mendelian randomisation pleiotropy residual sum and outlier (MR-PRESSO) approaches as sensitivity analyses to assess robustness of findings (20–22). Based on an intercept term, the MR-Egger approach can detect horizontal pleiotropy (20). The presence of pleiotropy was also evaluated applying the MR-PRESSO method. The MR-PRESSO method can remove outlying SNPs if present and re-evaluate the effect estimates (22). For evaluating the presence of heterogeneity between variant-specific estimates, we used the Cochran’s Q statistical test (22). We calculated statistical power using the method proposed by Brion and colleagues (23). Supplementary Table 2 shows the results for statistical power calculation. We carried out all MR analyses using the TwoSampleMR (version 0.5.6) and MR-PRESSO (version 1.0) packages in R version 4.1.0. Statistical significance was set at *P* < 0.05.

## Results

Among the SNPs used as instrumental variables, rs13198474, rs9296009, and rs931 have the strongest association with CD (*P* = 1.00 × 10^–200^). The 15 SNPs for CD corresponded to a *F*-statistic of 331.17, which explained approximately 17.0% of the variation in CD.

**FIGURE 1:**
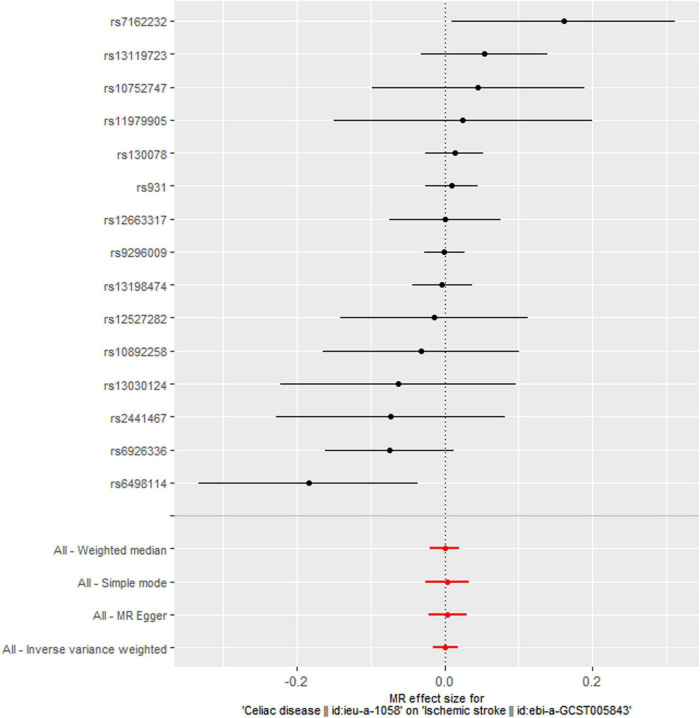
Meta-analytic mendelian randomization (MR) effect estimates and 95% confidence intervals for the association between celiac disease and ischemic stroke. The inverse variance weighted (IVW) method was applied in the main MR estimate. Sensitivity analyses were performed using the MR-Egger, weighted median, and simple mode methods.

The main MR analyses revealed no causal effect of genetically predicted CD on ischemic stroke (OR = 1.001, 95% CI: 0.984-1.018, *P* = 0.899), large artery stroke (OR = 1.003, 95% CI: 0.961-1.048, *P* = 0.876), cardioembolic stroke (OR = 1.009, 95% CI: 0.977-1.042, *P* = 0.568), small vessel stroke (OR = 1.023, 95% CI: 0.981-1.066, *P* = 0.298), coronary heart disease (OR = 0.995, 95% CI: 0.977-1.013, *P* = 0.596), myocardial infarction (OR = 0.994, 95% CI: 0.959-1.030, *P* = 0.736), angina (OR = 1.006, 95% CI: 0.981-1.032, *P* = 0.650), heart failure (OR = 0.999, 95% CI: 0.982-1.016, *P* = 0.879), atrial fibrillation (OR = 1.000, 95% CI: 0.990-1.011, *P* = 0.984), and venous thromboembolism (OR = 1.001, 95% CI: 0.971-1.032, *P* = 0.958) (Table 2 and Figure 1). Sensitivity analyses using the MR-Egger, weighted median, and simple mode methods also did not suggest a causal association between celiac disease and cardiovascular diseases (Table 2). The MR-Egger intercepts indicated no evidence of directional pleiotropy effects (all *P* > 0.100) (Table 3). In addition, the MR-PRESSO global test did not suggest the presence of pleiotropy (Table 3). The leave-one-out sensitivity analyses demonstrated that the causal estimates were not disproportionately affected by any single SNP (Supplementary Table 3).

**TABLE 2:** Assessing the cause effect of celiac disease on cardiovascular diseases.

Outcome	Number of instrumental SNPs	MR method	OR (95% CI)	*P*-value
				
Ischemic stroke	15	IVW	1.001 (0.984-1.018)	0.899
	15	MR-Egger	1.004 (0.979-1.030)	0.763
	15	Weighted median	1.000 (0.982-1.019)	0.961
	15	Simple mode	1.004 (0.978-1.031)	0.752
Ischemic stroke (large artery atherosclerosis)	15	IVW	1.003 (0.961-1.048)	0.876
	15	MR-Egger	1.040 (0.980-1.104)	0.222
	15	Weighted median	1.010 (0.963-1.060)	0.685
	15	Simple mode	0.956 (0.886-1.032)	0.272
Ischemic stroke (cardioembolic)	15	IVW	1.009 (0.977-1.042)	0.568
	15	MR-Egger	1.001 (0.955-1.051)	0.954
	15	Weighted median	1.024 (0.980-1.069)	0.291
	15	Simple mode	1.016 (0.948-1.090)	0.660
Ischemic stroke (small-vessel)	15	IVW	1.023 (0.981-1.066)	0.298
	15	MR-Egger	1.031 (0.970-1.096)	0.347
	15	Weighted median	1.017 (0.956-1.082)	0.592
	15	Simple mode	1.015 (0.917-1.124)	0.776
Coronary heart disease	15	IVW	0.995 (0.977-1.013)	0.596
	15	MR-Egger	0.983 (0.957-1.009)	0.229
	15	Weighted median	0.994 (0.973-1.014)	0.542
	15	Simple mode	1.002 (0.971-1.033)	0.906
Myocardial infarction	15	IVW	0.994 (0.959-1.030)	0.736
	15	MR-Egger	0.986 (0.935-1.041)	0.626
	15	Weighted median	0.984 (0.948-1.021)	0.380
	15	Simple mode	1.008 (0.937-1.084)	0.843
Angina	15	IVW	1.006 (0.981-1.032)	0.650
	15	MR-Egger	1.014 (0.975-1.054)	0.503
	15	Weighted median	1.009 (0.979-1.039)	0.562
	15	Simple mode	1.002 (0.961-1.045)	0.927
Heart failure	13	IVW	0.999 (0.982-1.016)	0.879
	13	MR-Egger	0.983 (0.959-1.008)	0.204
	13	Weighted median	0.993 (0.972-1.014)	0.519
	13	Simple mode	1.001 (0.970-1.034)	0.929
Atrial fibrillation	15	IVW	1.000 (0.990-1.011)	0.984
	15	MR-Egger	0.994 (0.979-1.009)	0.445
	15	Weighted median	0.996 (0.981-1.011)	0.588
	15	Simple mode	0.988 (0.965-1.011)	0.328
Venous thromboembolism	15	IVW	1.001 (0.971-1.032)	0.958
	15	MR-Egger	1.008 (0.963-1.057)	0.728
	15	Weighted median	0.989 (0.955-1.024)	0.521
	15	Simple mode	0.987 (0.958-1.040)	0.624

CI, confidence interval; IVW, inverse variance weighted; MR-Egger, Mendelian randomization Egger; OR, odds ratio.

**TABLE 3:** Evaluation for pleiotropy.

Outcome	MR-Egger intercept	*P*-value	MR-PRESSO global test *P*-value
Ischemic stroke	–0.001	0.762	0.313
Ischemic stroke (large artery atherosclerosis)	–0.019	0.131	0.758
Ischemic stroke (cardioembolic)	0.004	0.661	0.402
Ischemic stroke (small-vessel)	–0.004	0.721	0.502
Coronary heart disease	0.006	0.243	0.245
Myocardial infarction	0.004	0.713	0.055
Angina	–0.004	0.606	0.248
Heart failure	0.007	0.118	0.589
Atrial fibrillation	0.003	0.297	0.424
Venous thromboembolism	–0.004	0.670	0.155

MR, mendelian randomization.

For replication, we used a GWAS dataset built by Dubois et al. (24) for CD (GWAS ID: ieu-a-1060), including 3,796 cases and 8,154 controls of European ancestry. Seven instrumental SNPs were identified for CD using this dataset (Supplementary Table 4). Neither primary MR analyses nor sensitivity analyses indicated a causal effect of CD on ischemic stroke, large artery stroke, cardioembolic stroke, small vessel stroke, coronary heart disease, myocardial infarction, angina, heart failure, atrial fibrillation, and venous thromboembolism (Supplementary Table 5).

## Discussion

Although a number of observational studies have been performed to evaluate the relationship between CD and cardiovascular disease risk, whether CD have a causal effect on cardiovascular disease remains largely unknown. Our MR analyses provided no evidence to support a causal association of CD with ischemic stroke, large artery stroke, cardioembolic stroke, small vessel stroke, coronary heart disease, myocardial infarction, angina, heart failure, atrial fibrillation, and venous thromboembolism in individuals of European ancestry.

The findings of our study were concordant with those from several previous meta-analyses. Emilsson et al. (25) reviewed over 3,800 publications and selected nine studies in their meta-analyses for assessing the relationship between CD and cardiovascular outcomes including myocardial infarction and cardiovascular death. They found that CD was not associated with myocardial infarction (OR = 1.12, 95% CI: 0.83-1.40) and cardiovascular death (OR = 1.12, 95% CI: 0.96-1.29). Strengths of this meta-analysis included the use of systematic literature review as a methodology and eliminating the risk for miscounting study subjects. The major limitations were the low number of included publications and utilizing only data from individuals of European descent. A systematic review and meta-analysis by Heikkilä et al. (26) also did not provide evidence for supporting an association of CD with coronary heart disease and stroke. Their pooled estimates showed that the overall hazard ratio (HR) was 1.05 (95% CI: 0.93-1.19) for coronary heart disease, and the results were similar for stroke (HR: 1.09, 95% CI: 0.93-1.27) and brain hemorrhage (HR: 1.16, 95% CI: 0.97-1.37). Compared with other meta-analyses in the field, Heikkilä et al. (25) stated that the major strengths of their meta-analysis were the inclusion of studies with a prospective design and a low possibility of recall bias. By analyzing CD-associated genetic variants in a large meta-analytical dataset for coronary heart disease (22,233 cases and 64,762 controls), Jasen and colleagues (27) found that genetic variants associated with CD did not confer a higher risk of developing coronary heart disease. In addition, Jasen and colleagues suggested that the positive association between CD and coronary heart disease reported in observational studies may have been due to residual confounding. Their genetically based analysis did not support a causal contribution of CD itself to coronary heart disease risk.

The major strength of our study is the MR design. Compared with conventional observational studies, the possibility of residual confounding and reverse causation is greatly reduced in MR. Being a valuable tool for casual inference, MR has been widely used in the cardiovascular disease field (28, 29). We investigated the association of CD with 10 cardiovascular diseases using a two-sample MR design. Besides the primary MR analyses, a series of sensitivity analyses were conducted for ensuring the consistency and robustness of causal assessments. The MR-Egger intercept tests and MR-PRESSO global tests did not provide evidence of pleiotropy. Moreover, we used another GWAS dataset for CD for validating the MR findings for the null association of CD with the 10 cardiovascular diseases, yielding similar MR estimates.

### Limitations

Despite the strengths, some limitations of this study should be noted. Firstly, CD is a multifactorial autoimmune disease, and genetic factors only explain a small fraction of CD pathogenesis. In the GWAS dataset for CD, the extracted instrumental SNPs explained approximately 17.0% of the variation in CD, which was not high in MR analysis. However, *F*-statistics for all SNPs were larger than 10, greatly reducing the possibility that the null results were the result of weak instrument bias. Secondly, because the summary-level data did not provide information on gender, we could not evaluate the causal association of CD with cardiovascular diseases in males and females, respectively. Epidemiological studies suggested that gender may play a role in cardiovascular disease susceptibility and mortality (30, 31). Future large-scale MR studies using individual-level data are required to analyze gender-specific associations. Thirdly, the inverse variance weighted method can be subject to biases owing to sample overlap between the exposure and outcome datasets. We did not apply other MR methods such as MRlap that may account for bias introduced by sample overlap (32). Fourthly, our MR study was restricted to participants of European ancestry. This minimized population stratification bias, but the MR results could not be directly extrapolated to other ethnic groups.

## Conclusion

In summary, our findings suggest that CD itself has no causal effect on ischemic stroke, large artery stroke, cardioembolic stroke, small vessel stroke, coronary heart disease, myocardial infarction, angina, heart failure, atrial fibrillation, and venous thromboembolism in individuals of European ancestry. Well-conducted and long-term follow-up RCTs with large samples of well-characterized CD patients are needed to clarify our findings.

## Data availability statement

Publicly available datasets were analyzed in this study. This data can be found here: https://gwas.mrcieu.ac.uk/.

## Ethics statement

Ethical approval and written informed consent were not required, since we used only publicly available GWAS summary data in this Mendelian randomization study.

## Author contributions

JH contributed to study concept and design, acquisition and interpretation of data, statistical analyses, manuscript drafting, and revision.

## Footnotes

1
https://gwas.mrcieu.ac.uk/

2
https://github.com/MRCIEU/TwoSampleMR
