# Supplementary material for: Assessment of the causal association between celiac disease and cardiovascular diseases
Source: Front Cardiovasc Med. 2022 Oct 21;9:1017209. doi: 10.3389/fcvm.2022.1017209 (PMC9644835; doi:10.3389/fcvm.2022.1017209)
Supplement: Supplementary file 1 [file Table_1.docx]

Supplementary Table S1 Characteristics of the instruments for celiac disease and their associations with cardiovascular disease.

| **Exposure-Outcome** | **SNP** | **Chr** | **Position** | **EA** | **OA** | **Exposure effect** |  |  |  | **Outcome effect** |  |  |
| --- | --- | --- | --- | --- | --- | --- | --- | --- | --- | --- | --- | --- |
|  |  |  |  |  |  | **β** | **SE** | ***P*** |  | **β** | **SE** | ***P*** |
| CD-Ischemic stroke | rs10752747 | 1 | 2524915 | T | G | -0.116 | 0.020 | 5.05E-09 |  | -0.005 | 0.009 | 0.530 |
|  | rs10892258 | 11 | 118579865 | A | G | -0.150 | 0.022 | 1.73E-11 |  | 0.005 | 0.010 | 0.635 |
|  | rs11979905 | 7 | 37437877 | G | A | 0.163 | 0.029 | 2.49E-08 |  | 0.004 | 0.015 | 0.780 |
|  | rs12527282 | 6 | 137967252 | T | C | -0.154 | 0.021 | 1.69E-13 |  | 0.002 | 0.010 | 0.836 |
|  | rs12663317 | 6 | 32742827 | C | A | -0.616 | 0.043 | 3.53E-47 |  | -7.00E-04 | 0.024 | 0.977 |
|  | rs130078 | 6 | 31118565 | G | C | -0.577 | 0.024 | 3.35E-125 |  | -0.008 | 0.011 | 0.500 |
|  | rs13030124 | 2 | 204694263 | A | G | -0.105 | 0.019 | 2.40E-08 |  | 0.007 | 0.009 | 0.433 |
|  | rs13119723 | 4 | 123218313 | G | A | -0.309 | 0.028 | 7.60E-29 |  | -0.017 | 0.014 | 0.221 |
|  | rs13198474 | 6 | 25874423 | A | G | 0.946 | 0.031 | 1.00E-200 |  | -0.003 | 0.020 | 0.870 |
|  | rs2441467 | 2 | 61374149 | C | T | 0.105 | 0.019 | 1.70E-08 |  | -0.008 | 0.008 | 0.364 |
|  | rs6498114 | 16 | 10964118 | T | G | -0.131 | 0.021 | 5.83E-10 |  | 0.024 | 0.010 | 0.015 |
|  | rs6926336 | 6 | 32432464 | C | T | -0.812 | 0.080 | 6.12E-24 |  | 0.060 | 0.036 | 0.093 |
|  | rs7162232 | 15 | 75115895 | A | G | -0.117 | 0.020 | 7.97E-09 |  | -0.019 | 0.009 | 0.037 |
|  | rs9296009 | 6 | 32114515 | T | A | -0.954 | 0.029 | 1.00E-200 |  | 6.00E-04 | 0.013 | 0.962 |
|  | rs931 | 6 | 33054550 | A | G | 0.618 | 0.020 | 1.00E-200 |  | 0.006 | 0.011 | 0.603 |
| CD-Ischemic stroke (large artery atherosclerosis) | rs10752747 | 1 | 2524915 | T | G | -0.116 | 0.020 | 5.05E-09 |  | 0.026 | 0.021 | 0.210 |
|  | rs10892258 | 11 | 118579865 | A | G | -0.150 | 0.022 | 1.73E-11 |  | 0.013 | 0.023 | 0.577 |
|  | rs11979905 | 7 | 37437877 | G | A | 0.163 | 0.029 | 2.49E-08 |  | 0.077 | 0.036 | 0.031 |
|  | rs12527282 | 6 | 137967252 | T | C | -0.155 | 0.021 | 1.69E-13 |  | 0.040 | 0.024 | 0.090 |
|  | rs12663317 | 6 | 32742827 | C | A | -0.616 | 0.043 | 3.53E-47 |  | -0.018 | 0.056 | 0.744 |
|  | rs130078 | 6 | 31118565 | G | C | -0.577 | 0.024 | 3.35E-125 |  | 7.00E-04 | 0.025 | 0.978 |
|  | rs13030124 | 2 | 204694263 | A | G | -0.105 | 0.019 | 2.40E-08 |  | 0.036 | 0.021 | 0.083 |
|  | rs13119723 | 4 | 123218313 | G | A | -0.309 | 0.028 | 7.60E-29 |  | -0.011 | 0.033 | 0.732 |
|  | rs13198474 | 6 | 25874423 | A | G | 0.946 | 0.031 | 1.00E-200 |  | 0.106 | 0.049 | 0.033 |
|  | rs2441467 | 2 | 61374149 | C | T | 0.105 | 0.019 | 1.70E-08 |  | -0.012 | 0.020 | 0.561 |
|  | rs6498114 | 16 | 10964118 | T | G | -0.131 | 0.021 | 5.83E-10 |  | 0.016 | 0.023 | 0.494 |
|  | rs6926336 | 6 | 32432464 | C | T | -0.812 | 0.080 | 6.12E-24 |  | 0.073 | 0.092 | 0.431 |
|  | rs7162232 | 15 | 75115895 | A | G | -0.117 | 0.020 | 7.97E-09 |  | -0.019 | 0.022 | 0.370 |
|  | rs9296009 | 6 | 32114515 | T | A | -0.954 | 0.029 | 1.00E-200 |  | -0.022 | 0.030 | 0.466 |
|  | rs931 | 6 | 33054550 | A | G | 0.618 | 0.020 | 1.00E-200 |  | -0.029 | 0.025 | 0.238 |
| CD-Ischemic stroke (cardioembolic) | rs10752747 | 1 | 2524915 | T | G | -0.116 | 0.020 | 5.05E-09 |  | 0.003 | 0.018 | 0.851 |
|  | rs10892258 | 11 | 118579865 | A | G | -0.150 | 0.022 | 1.73E-11 |  | -0.003 | 0.020 | 0.887 |
|  | rs11979905 | 7 | 37437877 | G | A | 0.163 | 0.029 | 2.49E-08 |  | -0.001 | 0.029 | 0.965 |
|  | rs12527282 | 6 | 137967252 | T | C | -0.154 | 0.021 | 1.69E-13 |  | 0.018 | 0.021 | 0.386 |
|  | rs12663317 | 6 | 32742827 | C | A | -0.616 | 0.043 | 3.53E-47 |  | -0.019 | 0.045 | 0.668 |
|  | rs130078 | 6 | 31118565 | G | C | -0.577 | 0.024 | 3.35E-125 |  | -0.041 | 0.023 | 0.068 |
|  | rs13030124 | 2 | 204694263 | A | G | -0.105 | 0.019 | 2.40E-08 |  | -0.004 | 0.017 | 0.807 |
|  | rs13119723 | 4 | 123218313 | G | A | -0.309 | 0.028 | 7.60E-29 |  | -0.008 | 0.026 | 0.743 |
|  | rs13198474 | 6 | 25874423 | A | G | 0.946 | 0.031 | 1.00E-200 |  | -0.048 | 0.041 | 0.237 |
|  | rs2441467 | 2 | 61374149 | C | T | 0.105 | 0.019 | 1.70E-08 |  | 0.024 | 0.017 | 0.169 |
|  | rs6498114 | 16 | 10964118 | T | G | -0.131 | 0.021 | 5.83E-10 |  | 0.016 | 0.021 | 0.447 |
|  | rs6926336 | 6 | 32407322 | C | T | -0.812 | 0.080 | 6.12E-24 |  | 0.186 | 0.084 | 0.026 |
|  | rs7162232 | 15 | 75115895 | A | G | -0.117 | 0.020 | 7.97E-09 |  | -0.017 | 0.019 | 0.386 |
|  | rs9296009 | 6 | 32114515 | T | A | -0.954 | 0.029 | 1.00E-200 |  | -0.025 | 0.028 | 0.374 |
|  | rs931 | 6 | 33054550 | A | G | 0.618 | 0.020 | 1.00E-200 |  | -0.004 | 0.023 | 0.859 |
| CD-Ischemic stroke (small-vessel) | rs10752747 | 1 | 2524915 | T | G | -0.116 | 0.020 | 5.05E-09 |  | -0.002 | 0.024 | 0.919 |
|  | rs10892258 | 11 | 118579865 | A | G | -0.150 | 0.022 | 1.73E-11 |  | 0.033 | 0.026 | 0.203 |
|  | rs11979905 | 7 | 37437877 | G | A | 0.163 | 0.029 | 2.49E-08 |  | -0.011 | 0.037 | 0.771 |
|  | rs12527282 | 6 | 137967252 | T | C | -0.155 | 0.021 | 1.69E-13 |  | -0.019 | 0.026 | 0.450 |
|  | rs12663317 | 6 | 32742827 | C | A | -0.616 | 0.043 | 3.53E-47 |  | 0.018 | 0.057 | 0.755 |
|  | rs130078 | 6 | 31118565 | G | C | -0.577 | 0.024 | 3.35E-125 |  | 0.028 | 0.032 | 0.393 |
|  | rs13030124 | 2 | 204694263 | A | G | -0.105 | 0.019 | 2.40E-08 |  | -0.006 | 0.023 | 0.809 |
|  | rs13119723 | 4 | 123218313 | G | A | -0.309 | 0.028 | 7.60E-29 |  | -0.063 | 0.032 | 0.049 |
|  | rs13198474 | 6 | 25874423 | A | G | 0.946 | 0.031 | 1.00E-200 |  | 0.067 | 0.045 | 0.135 |
|  | rs2441467 | 2 | 61374149 | C | T | 0.105 | 0.019 | 1.70E-08 |  | -0.022 | 0.023 | 0.328 |
|  | rs6498114 | 16 | 10964118 | T | G | -0.131 | 0.021 | 5.83E-10 |  | 0.012 | 0.025 | 0.634 |
|  | rs6926336 | 6 | 32432464 | C | T | -0.812 | 0.080 | 6.12E-24 |  | 0.138 | 0.115 | 0.2306 |
|  | rs7162232 | 15 | 75115895 | A | G | -0.117 | 0.020 | 7.97E-09 |  | -0.027 | 0.026 | 0.297 |
|  | rs9296009 | 6 | 32114515 | T | A | -0.954 | 0.029 | 1.00E-200 |  | -0.011 | 0.038 | 0.780 |
|  | rs931 | 6 | 33054550 | A | G | 0.618 | 0.020 | 1.00E-200 |  | 0.042 | 0.034 | 0.211 |
| CD-Coronary heart disease | rs10752747 | 1 | 2524915 | T | G | -0.116 | 0.020 | 5.05E-09 |  | -0.008 | 0.010 | 0.376 |
|  | rs10892258 | 11 | 118579865 | A | G | -0.150 | 0.022 | 1.73E-11 |  | -0.003 | 0.011 | 0.756 |
|  | rs11979905 | 7 | 37437877 | G | A | 0.163 | 0.029 | 2.49E-08 |  | 0.035 | 0.016 | 0.025 |
|  | rs12527282 | 6 | 137967252 | T | C | -0.154 | 0.021 | 1.69E-13 |  | -0.009 | 0.011 | 0.380 |
|  | rs12663317 | 6 | 32742827 | C | A | -0.616 | 0.043 | 3.53E-47 |  | 0.014 | 0.018 | 0.442 |
|  | rs130078 | 6 | 31118565 | G | C | -0.577 | 0.024 | 3.35E-125 |  | 0.019 | 0.011 | 0.085 |
|  | rs13030124 | 2 | 204694263 | A | G | -0.105 | 0.019 | 2.40E-08 |  | 0.013 | 0.010 | 0.178 |
|  | rs13119723 | 4 | 123218313 | G | A | -0.309 | 0.028 | 7.60E-29 |  | -0.002 | 0.014 | 0.880 |
|  | rs13198474 | 6 | 25874423 | A | G | 0.946 | 0.031 | 1.00E-200 |  | -0.006 | 0.022 | 0.794 |
|  | rs2441467 | 2 | 61374149 | C | T | 0.105 | 0.019 | 1.70E-08 |  | -0.008 | 0.010 | 0.432 |
|  | rs6498114 | 16 | 10964118 | T | G | -0.131 | 0.021 | 5.83E-10 |  | -0.012 | 0.011 | 0.258 |
|  | rs6926336 | 6 | 32432464 | C | T | -0.812 | 0.080 | 6.12E-24 |  | -0.069 | 0.040 | 0.086 |
|  | rs7162232 | 15 | 75115895 | A | G | -0.117 | 0.020 | 7.97E-09 |  | -0.014 | 0.010 | 0.171 |
|  | rs9296009 | 6 | 32114515 | T | A | -0.954 | 0.029 | 1.00E-200 |  | 0.006 | 0.014 | 0.648 |
|  | rs931 | 6 | 33054550 | A | G | 0.618 | 0.020 | 1.00E-200 |  | -0.003 | 0.012 | 0.777 |
| CD-Myocardial infarction | rs10752747 | 1 | 2524915 | T | G | -0.116 | 0.020 | 5.05E-09 |  | 0.008 | 0.017 | 0.624 |
|  | rs10892258 | 11 | 118579865 | A | G | -0.150 | 0.022 | 1.73E-11 |  | 0.011 | 0.019 | 0.573 |
|  | rs11979905 | 7 | 37437877 | G | A | 0.163 | 0.029 | 2.49E-08 |  | 0.052 | 0.025 | 0.039 |
|  | rs12527282 | 6 | 137967252 | T | C | -0.154 | 0.021 | 1.69E-13 |  | 0.024 | 0.020 | 0.213 |
|  | rs12663317 | 6 | 32742827 | C | A | -0.616 | 0.043 | 3.53E-47 |  | -0.028 | 0.028 | 0.324 |
|  | rs130078 | 6 | 31118565 | G | C | -0.577 | 0.024 | 3.35E-125 |  | 0.046 | 0.019 | 0.014 |
|  | rs13030124 | 2 | 204694263 | A | G | -0.105 | 0.019 | 2.40E-08 |  | 0.024 | 0.017 | 0.165 |
|  | rs13119723 | 4 | 123218313 | G | A | -0.309 | 0.028 | 7.60E-29 |  | -0.050 | 0.026 | 0.051 |
|  | rs13198474 | 6 | 25874423 | A | G | 0.946 | 0.031 | 1.00E-200 |  | 0.036 | 0.039 | 0.353 |
|  | rs2441467 | 2 | 61374149 | C | T | 0.105 | 0.019 | 1.70E-08 |  | 0.006 | 0.016 | 0.733 |
|  | rs6498114 | 16 | 10964118 | T | G | -0.131 | 0.021 | 5.83E-10 |  | -0.018 | 0.018 | 0.316 |
|  | rs6926336 | 6 | 32407322 | C | T | -0.812 | 0.080 | 6.12E-24 |  | -0.067 | 0.049 | 0.173 |
|  | rs7162232 | 15 | 75115895 | A | G | -0.117 | 0.020 | 7.97E-09 |  | -0.023 | 0.017 | 0.179 |
|  | rs9296009 | 6 | 32114515 | T | A | -0.954 | 0.029 | 1.00E-200 |  | 0.024 | 0.022 | 0.290 |
|  | rs931 | 6 | 33054550 | A | G | 0.618 | 0.020 | 1.00E-200 |  | -0.006 | 0.019 | 0.760 |
| CD-Angina | rs10752747 | 1 | 2524915 | T | G | -0.116 | 0.020 | 5.05E-09 |  | 0.018 | 0.014 | 0.214 |
|  | rs10892258 | 11 | 118579865 | A | G | -0.150 | 0.022 | 1.73E-11 |  | 0.023 | 0.016 | 0.153 |
|  | rs11979905 | 7 | 37437877 | G | A | 0.163 | 0.029 | 2.49E-08 |  | 0.044 | 0.021 | 0.035 |
|  | rs12527282 | 6 | 137967252 | T | C | -0.155 | 0.021 | 1.69E-13 |  | 9.00E-04 | 0.017 | 0.957 |
|  | rs12663317 | 6 | 32742827 | C | A | -0.616 | 0.043 | 3.53E-47 |  | -0.006 | 0.024 | 0.796 |
|  | rs130078 | 6 | 31118565 | G | C | -0.577 | 0.024 | 3.35E-125 |  | 0.023 | 0.016 | 0.149 |
|  | rs13030124 | 2 | 204694263 | A | G | -0.105 | 0.019 | 2.40E-08 |  | 0.004 | 0.015 | 0.792 |
|  | rs13119723 | 4 | 123218313 | G | A | -0.309 | 0.028 | 7.60E-29 |  | -0.029 | 0.022 | 0.182 |
|  | rs13198474 | 6 | 25874423 | A | G | 0.946 | 0.031 | 1.00E-200 |  | 0.0161 | 0.034 | 0.631 |
|  | rs2441467 | 2 | 61374149 | C | T | 0.105 | 0.019 | 1.70E-08 |  | -0.003 | 0.014 | 0.830 |
|  | rs6498114 | 16 | 10964118 | T | G | -0.131 | 0.021 | 5.83E-10 |  | 0.023 | 0.015 | 0.137 |
|  | rs6926336 | 6 | 32407322 | C | T | -0.812 | 0.080 | 6.12E-24 |  | -0.035 | 0.042 | 0.406 |
|  | rs7162232 | 15 | 75115895 | A | G | -0.117 | 0.020 | 7.97E-09 |  | -0.023 | 0.015 | 0.115 |
|  | rs9296009 | 6 | 32114515 | T | A | -0.954 | 0.029 | 1.00E-200 |  | -0.007 | 0.019 | 0.728 |
|  | rs931 | 6 | 33054550 | A | G | 0.618 | 0.020 | 1.00E-200 |  | 0.016 | 0.016 | 0.331 |
| CD-Heart failure | rs10752747 | 1 | 2524915 | T | G | -0.116 | 0.020 | 5.05E-09 |  | 0.0061 | 0.009 | 0.474 |
|  | rs10892258 | 11 | 118579865 | A | G | -0.150 | 0.022 | 1.73E-11 |  | 0.0068 | 0.009 | 0.463 |
|  | rs11979905 | 7 | 37437877 | G | A | 0.163 | 0.029 | 2.49E-08 |  | 0.018 | 0.013 | 0.158 |
|  | rs12527282 | 6 | 137967252 | T | C | -0.154 | 0.021 | 1.69E-13 |  | -0.0013 | 0.009 | 0.886 |
|  | rs12663317 | 6 | 32742827 | C | A | -0.616 | 0.043 | 3.53E-47 |  | -0.0025 | 0.015 | 0.864 |
|  | rs13030124 | 2 | 204694263 | A | G | -0.105 | 0.019 | 2.40E-08 |  | -0.0061 | 0.008 | 0.447 |
|  | rs13119723 | 4 | 123218313 | G | A | -0.309 | 0.028 | 7.60E-29 |  | -0.0082 | 0.010 | 0.430 |
|  | rs13198474 | 6 | 25874423 | A | G | 0.946 | 0.031 | 1.00E-200 |  | -0.0033 | 0.016 | 0.837 |
|  | rs2441467 | 2 | 61374149 | C | T | 0.105 | 0.019 | 1.70E-08 |  | 0.0123 | 0.008 | 0.115 |
|  | rs6498114 | 16 | 10964118 | T | G | -0.131 | 0.021 | 5.83E-10 |  | -0.0101 | 0.009 | 0.272 |
|  | rs6926336 | 6 | 32432464 | C | T | -0.812 | 0.080 | 6.12E-24 |  | 0.008 | 0.027 | 0.769 |
|  | rs7162232 | 15 | 75115895 | A | G | -0.117 | 0.020 | 7.97E-09 |  | -0.0053 | 0.009 | 0.554 |
|  | rs931 | 6 | 33054550 | A | G | 0.618 | 0.020 | 1.00E-200 |  | -0.0102 | 0.009 | 0.249 |
| CD-Atrial fibrillation | rs10752747 | 1 | 2524915 | T | G | -0.116 | 0.020 | 5.05E-09 |  | -1.00E-04 | 0.007 | 0.992 |
|  | rs10892258 | 11 | 118579865 | A | G | -0.150 | 0.022 | 1.73E-11 |  | 0.001 | 0.008 | 0.894 |
|  | rs11979905 | 7 | 37437877 | G | A | 0.163 | 0.029 | 2.49E-08 |  | -0.004 | 0.011 | 0.719 |
|  | rs12527282 | 6 | 137967252 | T | C | -0.155 | 0.021 | 1.69E-13 |  | -0.009 | 0.008 | 0.228 |
|  | rs12663317 | 6 | 32742827 | C | A | -0.616 | 0.043 | 3.53E-47 |  | -0.027 | 0.014 | 0.046 |
|  | rs130078 | 6 | 31118565 | G | C | -0.577 | 0.024 | 3.35E-125 |  | 0.002 | 0.008 | 0.766 |
|  | rs13030124 | 2 | 204694263 | A | G | -0.105 | 0.019 | 2.40E-08 |  | -0.008 | 0.007 | 0.221 |
|  | rs13119723 | 4 | 123218313 | G | A | -0.309 | 0.028 | 7.60E-29 |  | 0.005 | 0.009 | 0.601 |
|  | rs13198474 | 6 | 25874423 | A | G | 0.946 | 0.031 | 1.00E-200 |  | -0.013 | 0.013 | 0.2979 |
|  | rs2441467 | 2 | 61374149 | C | T | 0.105 | 0.019 | 1.70E-08 |  | 0.010 | 0.007 | 0.141 |
|  | rs6498114 | 16 | 10964118 | T | G | -0.131 | 0.021 | 5.83E-10 |  | 0.002 | 0.008 | 0.779 |
|  | rs6926336 | 6 | 32432464 | C | T | -0.812 | 0.080 | 6.12E-24 |  | 0.033 | 0.027 | 0.219 |
|  | rs7162232 | 15 | 75115895 | A | G | -0.117 | 0.020 | 7.97E-09 |  | 0.002 | 0.007 | 0.839 |
|  | rs9296009 | 6 | 32114515 | T | A | -0.954 | 0.029 | 1.00E-200 |  | -0.008 | 0.009 | 0.374 |
|  | rs931 | 6 | 33054550 | A | G | 0.618 | 0.020 | 1.00E-200 |  | -0.008 | 0.008 | 0.329 |
| CD-Venous thromboembolism | rs10752747 | 1 | 2524915 | T | G | -0.116 | 0.020 | 5.05E-09 |  | 0.022 | 0.017 | 0.181 |
|  | rs10892258 | 11 | 118579865 | A | G | -0.150 | 0.022 | 1.73E-11 |  | 0.007 | 0.019 | 0.721 |
|  | rs11979905 | 7 | 37437877 | G | A | 0.163 | 0.029 | 2.49E-08 |  | -0.031 | 0.024 | 0.207 |
|  | rs12527282 | 6 | 137967252 | T | C | -0.155 | 0.021 | 1.69E-13 |  | 0.036 | 0.019 | 0.062 |
|  | rs12663317 | 6 | 32742827 | C | A | -0.616 | 0.043 | 3.53E-47 |  | 0.007 | 0.028 | 0.802 |
|  | rs130078 | 6 | 31118565 | G | C | -0.577 | 0.024 | 3.35E-125 |  | -0.043 | 0.018 | 0.018 |
|  | rs13030124 | 2 | 204694263 | A | G | -0.105 | 0.019 | 2.40E-08 |  | -0.017 | 0.017 | 0.319 |
|  | rs13119723 | 4 | 123218313 | G | A | -0.309 | 0.028 | 7.60E-29 |  | -0.010 | 0.025 | 0.701 |
|  | rs13198474 | 6 | 25874423 | A | G | 0.946 | 0.031 | 1.00E-200 |  | -0.046 | 0.038 | 0.230 |
|  | rs2441467 | 2 | 61374149 | C | T | 0.105 | 0.019 | 1.70E-08 |  | -0.003 | 0.016 | 0.834 |
|  | rs6498114 | 16 | 10964118 | T | G | -0.131 | 0.021 | 5.83E-10 |  | -0.032 | 0.018 | 0.065 |
|  | rs6926336 | 6 | 32407322 | C | T | -0.812 | 0.080 | 6.12E-24 |  | -0.040 | 0.048 | 0.403 |
|  | rs7162232 | 15 | 75115895 | A | G | -0.117 | 0.020 | 7.97E-09 |  | 0.014 | 0.017 | 0.421 |
|  | rs9296009 | 6 | 32114515 | T | A | -0.954 | 0.029 | 1.00E-200 |  | 0.011 | 0.022 | 0.615 |
|  | rs931 | 6 | 33054550 | A | G | 0.618 | 0.020 | 1.00E-200 |  | -0.004 | 0.019 | 0.813 |

SNP, single nucleotide polymorphism; SE, standard error.
