# Supplementary material for: Assessment of the causal association between celiac disease and cardiovascular diseases
Source: Front Cardiovasc Med. 2022 Oct 21;9:1017209. doi: 10.3389/fcvm.2022.1017209 (PMC9644835; doi:10.3389/fcvm.2022.1017209)
Supplement: Supplementary file 2 [file Table_2.docx]

Supplementary Table S2 Power calculation.

| Outcome | Cases/controls | OR ≤ | OR ≥ |
| --- | --- | --- | --- |
| Ischemic stroke | 34217/406111 | 0.962 | 1.038 |
| Ischemic stroke (large artery atherosclerosis) | 4373/406111 | 0.897 | 1.103 |
| Ischemic stroke (cardioembolic) | 7193/406111 | 0.919 | 1.081 |
| Ischemic stroke (small-vessel) | 5386/192662 | 0.906 | 1.094 |
| Coronary heart disease | 22233/64762 | 0.948 | 1.053 |
| Myocardial infarction | 11622/187840 | 0.935 | 1.065 |
| Angina | 18168/187840 | 0.948 | 1.053 |
| Heart failure | 47309/930014 | 0.968 | 1.032 |
| Atrial fibrillation | 60620/970216 | 0.972 | 1.028 |
| Venous thromboembolism | 9176/209616 | 0.928 | 1.073 |

ORs were calculated by setting 80% of power.
