# Supplementary material for: Assessment of the causal association between celiac disease and cardiovascular diseases
Source: Front Cardiovasc Med. 2022 Oct 21;9:1017209. doi: 10.3389/fcvm.2022.1017209 (PMC9644835; doi:10.3389/fcvm.2022.1017209)
Supplement: Supplementary file 3 [file Table_3.docx]

Supplementary Table S3 Leave-one-out analyses using the IVW method.

| Outcome |  | SNP | OR | 95% lower confidence limit | 95% confidence upper limit |
| --- | --- | --- | --- | --- | --- |
| Ischemic stroke |  | All | 1.001 | 0.984 | 1.018 |
|  | Removing | rs10752747 | 1.001 | 0.983 | 1.018 |
|  | Removing | rs10892258 | 1.002 | 0.984 | 1.020 |
|  | Removing | rs11979905 | 1.001 | 0.983 | 1.019 |
|  | Removing | rs12527282 | 1.001 | 0.984 | 1.019 |
|  | Removing | rs12663317 | 1.001 | 0.983 | 1.020 |
|  | Removing | rs130078 | 0.999 | 0.980 | 1.018 |
|  | Removing | rs13030124 | 1.002 | 0.984 | 1.019 |
|  | Removing | rs13119723 | 0.999 | 0.982 | 1.017 |
|  | Removing | rs13198474 | 1.002 | 0.983 | 1.021 |
|  | Removing | rs2441467 | 1.002 | 0.985 | 1.019 |
|  | Removing | rs6498114 | 1.003 | 0.988 | 1.019 |
|  | Removing | rs6926336 | 1.004 | 0.987 | 1.020 |
|  | Removing | rs7162232 | 0.999 | 0.984 | 1.015 |
|  | Removing | rs9296009 | 1.002 | 0.981 | 1.024 |
|  | Removing | rs931 | 0.999 | 0.980 | 1.019 |
| Ischemic stroke (large artery atherosclerosis) |  | All | 1.003 | 0.961 | 1.048 |
|  | Removing | rs10752747 | 1.006 | 0.963 | 1.050 |
|  | Removing | rs10892258 | 1.005 | 0.961 | 1.051 |
|  | Removing | rs11979905 | 1.000 | 0.961 | 1.041 |
|  | Removing | rs12527282 | 1.007 | 0.966 | 1.050 |
|  | Removing | rs12663317 | 1.002 | 0.958 | 1.049 |
|  | Removing | rs130078 | 1.004 | 0.956 | 1.056 |
|  | Removing | rs13030124 | 1.006 | 0.965 | 1.049 |
|  | Removing | rs13119723 | 1.002 | 0.958 | 1.049 |
|  | Removing | rs13198474 | 0.989 | 0.948 | 1.032 |
|  | Removing | rs2441467 | 1.004 | 0.961 | 1.050 |
|  | Removing | rs6498114 | 1.005 | 0.961 | 1.051 |
|  | Removing | rs6926336 | 1.006 | 0.962 | 1.052 |
|  | Removing | rs7162232 | 1.002 | 0.958 | 1.047 |
|  | Removing | rs9296009 | 0.994 | 0.942 | 1.049 |
|  | Removing | rs931 | 1.017 | 0.969 | 1.066 |
| Ischemic stroke (cardioembolic) |  | All | 1.009 | 0.977 | 1.042 |
|  | Removing | rs10752747 | 1.010 | 0.977 | 1.044 |
|  | Removing | rs10892258 | 1.009 | 0.976 | 1.044 |
|  | Removing | rs11979905 | 1.010 | 0.976 | 1.044 |
|  | Removing | rs12527282 | 1.011 | 0.979 | 1.045 |
|  | Removing | rs12663317 | 1.008 | 0.974 | 1.043 |
|  | Removing | rs130078 | 0.997 | 0.963 | 1.032 |
|  | Removing | rs13030124 | 1.009 | 0.976 | 1.043 |
|  | Removing | rs13119723 | 1.009 | 0.975 | 1.044 |
|  | Removing | rs13198474 | 1.019 | 0.985 | 1.055 |
|  | Removing | rs2441467 | 1.007 | 0.976 | 1.040 |
|  | Removing | rs6498114 | 1.011 | 0.978 | 1.044 |
|  | Removing | rs6926336 | 1.015 | 0.984 | 1.048 |
|  | Removing | rs7162232 | 1.008 | 0.976 | 1.042 |
|  | Removing | rs9296009 | 1.002 | 0.964 | 1.042 |
|  | Removing | rs931 | 1.013 | 0.977 | 1.051 |
| Ischemic stroke (small-vessel) |  | All | 1.023 | 0.981 | 1.066 |
|  | Removing | rs10752747 | 1.023 | 0.979 | 1.068 |
|  | Removing | rs10892258 | 1.026 | 0.984 | 1.071 |
|  | Removing | rs11979905 | 1.023 | 0.980 | 1.068 |
|  | Removing | rs12527282 | 1.021 | 0.978 | 1.065 |
|  | Removing | rs12663317 | 1.025 | 0.982 | 1.071 |
|  | Removing | rs130078 | 1.035 | 0.989 | 1.083 |
|  | Removing | rs13030124 | 1.022 | 0.979 | 1.067 |
|  | Removing | rs13119723 | 1.014 | 0.972 | 1.059 |
|  | Removing | rs13198474 | 1.010 | 0.964 | 1.059 |
|  | Removing | rs2441467 | 1.025 | 0.983 | 1.069 |
|  | Removing | rs6498114 | 1.024 | 0.981 | 1.068 |
|  | Removing | rs6926336 | 1.027 | 0.984 | 1.072 |
|  | Removing | rs7162232 | 1.021 | 0.978 | 1.064 |
|  | Removing | rs9296009 | 1.027 | 0.976 | 1.080 |
|  | Removing | rs931 | 1.014 | 0.969 | 1.061 |
| Coronary heart disease |  | All | 0.995 | 0.977 | 1.013 |
|  | Removing | rs10752747 | 0.994 | 0.976 | 1.013 |
|  | Removing | rs10892258 | 0.995 | 0.976 | 1.014 |
|  | Removing | rs11979905 | 0.994 | 0.976 | 1.010 |
|  | Removing | rs12527282 | 0.994 | 0.976 | 1.013 |
|  | Removing | rs12663317 | 0.996 | 0.977 | 1.016 |
|  | Removing | rs130078 | 1.001 | 0.982 | 1.021 |
|  | Removing | rs13030124 | 0.996 | 0.978 | 1.015 |
|  | Removing | rs13119723 | 0.995 | 0.976 | 1.014 |
|  | Removing | rs13198474 | 0.995 | 0.975 | 1.016 |
|  | Removing | rs2441467 | 0.996 | 0.977 | 1.014 |
|  | Removing | rs6498114 | 0.994 | 0.976 | 1.012 |
|  | Removing | rs6926336 | 0.993 | 0.975 | 1.010 |
|  | Removing | rs7162232 | 0.994 | 0.976 | 1.012 |
|  | Removing | rs9296009 | 0.996 | 0.973 | 1.019 |
|  | Removing | rs931 | 0.995 | 0.974 | 1.016 |
| Myocardial infarction |  | All | 0.994 | 0.959 | 1.030 |
|  | Removing | rs10752747 | 0.994 | 0.959 | 1.032 |
|  | Removing | rs10892258 | 0.995 | 0.959 | 1.032 |
|  | Removing | rs11979905 | 0.991 | 0.959 | 1.025 |
|  | Removing | rs12527282 | 0.996 | 0.961 | 1.032 |
|  | Removing | rs12663317 | 0.989 | 0.953 | 1.027 |
|  | Removing | rs130078 | 1.009 | 0.974 | 1.045 |
|  | Removing | rs13030124 | 0.995 | 0.961 | 1.031 |
|  | Removing | rs13119723 | 0.990 | 0.956 | 1.024 |
|  | Removing | rs13198474 | 0.989 | 0.952 | 1.027 |
|  | Removing | rs2441467 | 0.993 | 0.958 | 1.031 |
|  | Removing | rs6498114 | 0.993 | 0.957 | 1.029 |
|  | Removing | rs6926336 | 0.989 | 0.955 | 1.026 |
|  | Removing | rs7162232 | 0.992 | 0.958 | 1.028 |
|  | Removing | rs9296009 | 1.003 | 0.960 | 1.047 |
|  | Removing | rs931 | 0.995 | 0.955 | 1.036 |
| Angina |  | All | 1.006 | 0.981 | 1.032 |
|  | Removing | rs10752747 | 1.007 | 0.982 | 1.033 |
|  | Removing | rs10892258 | 1.008 | 0.983 | 1.033 |
|  | Removing | rs11979905 | 1.004 | 0.981 | 1.027 |
|  | Removing | rs12527282 | 1.006 | 0.980 | 1.033 |
|  | Removing | rs12663317 | 1.006 | 0.978 | 1.034 |
|  | Removing | rs130078 | 1.015 | 0.989 | 1.042 |
|  | Removing | rs13030124 | 1.006 | 0.980 | 1.033 |
|  | Removing | rs13119723 | 1.004 | 0.978 | 1.030 |
|  | Removing | rs13198474 | 1.005 | 0.977 | 1.033 |
|  | Removing | rs2441467 | 1.006 | 0.980 | 1.033 |
|  | Removing | rs6498114 | 1.008 | 0.983 | 1.033 |
|  | Removing | rs6926336 | 1.004 | 0.978 | 1.031 |
|  | Removing | rs7162232 | 1.004 | 0.980 | 1.030 |
|  | Removing | rs9296009 | 1.005 | 0.974 | 1.038 |
|  | Removing | rs931 | 1.001 | 0.973 | 1.031 |
| Heart failure |  | All | 0.999 | 0.982 | 1.016 |
|  | Removing | rs10752747 | 0.999 | 0.983 | 1.016 |
|  | Removing | rs10892258 | 1.000 | 0.983 | 1.017 |
|  | Removing | rs11979905 | 0.997 | 0.981 | 1.014 |
|  | Removing | rs12527282 | 0.999 | 0.982 | 1.016 |
|  | Removing | rs12663317 | 0.998 | 0.980 | 1.016 |
|  | Removing | rs13030124 | 0.998 | 0.981 | 1.015 |
|  | Removing | rs13119723 | 0.997 | 0.980 | 1.014 |
|  | Removing | rs13198474 | 0.999 | 0.980 | 1.019 |
|  | Removing | rs2441467 | 0.997 | 0.980 | 1.014 |
|  | Removing | rs6498114 | 0.998 | 0.981 | 1.015 |
|  | Removing | rs6926336 | 0.999 | 0.982 | 1.017 |
|  | Removing | rs7162232 | 0.998 | 0.981 | 1.015 |
|  | Removing | rs931 | 1.007 | 0.986 | 1.029 |
| Atrial fibrillation |  | All | 1.000 | 0.990 | 1.011 |
|  | Removing | rs10752747 | 1.000 | 0.989 | 1.011 |
|  | Removing | rs10892258 | 1.000 | 0.989 | 1.011 |
|  | Removing | rs11979905 | 1.000 | 0.989 | 1.010 |
|  | Removing | rs12527282 | 0.999 | 0.987 | 1.008 |
|  | Removing | rs12663317 | 0.997 | 0.989 | 1.013 |
|  | Removing | rs130078 | 1.001 | 0.989 | 1.010 |
|  | Removing | rs13030124 | 1.000 | 0.990 | 1.012 |
|  | Removing | rs13119723 | 1.001 | 0.991 | 1.014 |
|  | Removing | rs13198474 | 1.003 | 0.989 | 1.010 |
|  | Removing | rs2441467 | 0.999 | 0.989 | 1.011 |
|  | Removing | rs6498114 | 1.000 | 0.991 | 1.012 |
|  | Removing | rs6926336 | 1.001 | 0.989 | 1.011 |
|  | Removing | rs7162232 | 1.000 | 0.984 | 1.009 |
|  | Removing | rs9296009 | 0.996 | 0.991 | 1.014 |
|  | Removing | rs931 | 1.003 | 0.989 | 1.011 |
| Venous thromboembolism |  | All | 1.001 | 0.971 | 1.032 |
|  | Removing | rs10752747 | 1.002 | 0.972 | 1.033 |
|  | Removing | rs10892258 | 1.001 | 0.970 | 1.034 |
|  | Removing | rs11979905 | 1.002 | 0.972 | 1.033 |
|  | Removing | rs12527282 | 1.003 | 0.975 | 1.033 |
|  | Removing | rs12663317 | 1.002 | 0.969 | 1.036 |
|  | Removing | rs130078 | 0.986 | 0.958 | 1.015 |
|  | Removing | rs13030124 | 1.000 | 0.969 | 1.031 |
|  | Removing | rs13119723 | 1.000 | 0.969 | 1.033 |
|  | Removing | rs13198474 | 1.007 | 0.975 | 1.039 |
|  | Removing | rs2441467 | 1.001 | 0.970 | 1.033 |
|  | Removing | rs6498114 | 0.998 | 0.970 | 1.028 |
|  | Removing | rs6926336 | 0.998 | 0.967 | 1.031 |
|  | Removing | rs7162232 | 1.002 | 0.971 | 1.034 |
|  | Removing | rs9296009 | 1.007 | 0.969 | 1.046 |
|  | Removing | rs931 | 1.003 | 0.968 | 1.038 |

SNP, single nucleotide polymorphism; OR, odds ratio; IVW, inverse variance weighted.
