# Supplementary material for: Assessment of the causal association between celiac disease and cardiovascular diseases
Source: Front Cardiovasc Med. 2022 Oct 21;9:1017209. doi: 10.3389/fcvm.2022.1017209 (PMC9644835; doi:10.3389/fcvm.2022.1017209)
Supplement: Supplementary file 4 [file Table_4.docx]

Supplementary Table S4 Characteristics of the instruments for celiac disease using the GWAS dataset of ieu-a-1060.

| **SNP** | **Chr** | **Position** | **EA** | **OA** | **Exposure effect** |  |  |
| --- | --- | --- | --- | --- | --- | --- | --- |
|  |  |  |  |  | **β** | **SE** | ***P*** |
| rs11221335 | 11 | 128385906 | C | T | 0.218 | 0.033 | 4.16E-11 |
| rs13091753 | 3 | 188114589 | T | G | 0.233 | 0.029 | 2.38E-16 |
| rs13132933 | 4 | 123010587 | C | T | -0.275 | 0.040 | 7.85E-12 |
| rs1323292 | 1 | 192541021 | A | G | 0.234 | 0.040 | 4.02E-09 |
| rs1468791 | 2 | 103092021 | G | A | -0.223 | 0.033 | 1.66E-11 |
| rs6933404 | 6 | 137959235 | C | T | 0.241 | 0.034 | 6.16E-13 |
| rs9819226 | 3 | 159654171 | C | T | -0.177 | 0.028 | 4.60E-10 |

SNP, single nucleotide polymorphism; SE, standard error; GWAS, genome-wide association study.
