# Supplementary material for: Assessment of the causal association between celiac disease and cardiovascular diseases
Source: Front Cardiovasc Med. 2022 Oct 21;9:1017209. doi: 10.3389/fcvm.2022.1017209 (PMC9644835; doi:10.3389/fcvm.2022.1017209)
Supplement: Supplementary file 5 [file Table_5.docx]

Supplementary Table S5 The cause effect of celiac disease on cardiovascular diseases using the GWAS dataset of ieu-a-1060.

| Outcome | Number of instrumental SNPs | MR method | OR (95% CI) | *P*-value |
| --- | --- | --- | --- | --- |
|  |  |  |  |  |
| Ischemic stroke | 7 | IVW | 0.989 (0.957-1.022) | 0.517 |
|  | 7 | MR-Egger | 1.197 (0.917-1.563) | 0.244 |
|  | 7 | Weighted median | 1.000 (0.959-1.044) | 0.990 |
|  | 7 | Simple mode | 1.009 (0.945-1.078) | 0.791 |
| Ischemic stroke (large artery atherosclerosis) | 7 | IVW | 1.020 (0.914-1.139) | 0.722 |
|  | 7 | MR-Egger | 1.114 (0.416-2.980) | 0.838 |
|  | 7 | Weighted median | 1.038 (0.927-1.163) | 0.516 |
|  | 7 | Simple mode | 1.006 (0.836-1.210) | 0.955 |
| Ischemic stroke (cardioembolic) | 7 | IVW | 1.014 (0.923-1.113) | 0.775 |
|  | 7 | MR-Egger | 1.293 (0.581-2.877) | 0.557 |
|  | 7 | Weighted median | 1.011 (0.914-1.118) | 0.836 |
|  | 7 | Simple mode | 0.936 (0.780-1.124) | 0.505 |
| Ischemic stroke (small-vessel) | 7 | IVW | 1.034 (0.948-1.128) | 0.454 |
|  | 7 | MR-Egger | 1.630 (0.806-3.298) | 0.232 |
|  | 7 | Weighted median | 1.039 (0.927-1.164) | 0.509 |
|  | 7 | Simple mode | 1.092 (0.911-1.309) | 0.378 |
| Coronary heart disease | 7 | IVW | 1.041 (0.998-1.085) | 0.061 |
|  | 7 | MR-Egger | 1.104 (0.761-1.603) | 0.624 |
|  | 7 | Weighted median | 1.018 (0.971-1.068) | 0.467 |
|  | 7 | Simple mode | 1.013 (0.940-1.091) | 0.755 |
| Myocardial infarction | 7 | IVW | 1.021 (0.958-1.088) | 0.523 |
|  | 7 | MR-Egger | 1.267 (0.753-2.132) | 0.414 |
|  | 7 | Weighted median | 1.032 (0.951-1.120) | 0.447 |
|  | 7 | Simple mode | 1.034 (0.917-1.167) | 0.602 |
| Angina | 7 | IVW | 1.011 (0.950-1.076) | 0.738 |
|  | 7 | MR-Egger | 1.064 (0.612-1.852) | 0.835 |
|  | 7 | Weighted median | 1.001 (0.930-1.078) | 0.971 |
|  | 7 | Simple mode | 0.995 (0.893-1.107) | 0.874 |
| Heart failure | 7 | IVW | 1.003 (0.964-1.043) | 0.894 |
|  | 7 | MR-Egger | 1.111 (0.795-1.552) | 0.566 |
|  | 7 | Weighted median | 0.986 (0.946-1.028) | 0.510 |
|  | 7 | Simple mode | 0.978 (0.923-1.040) | 0.539 |
| Atrial fibrillation | 7 | IVW | 1.032 (0.989-1.076) | 0.147 |
|  | 7 | MR-Egger | 1.131 (0.784-1.632) | 0.539 |
|  | 7 | Weighted median | 1.037 (0.998-1.077) | 0.060 |
|  | 7 | Simple mode | 1.042 (0.971-1.119) | 0.294 |
| Venous thromboembolism | 7 | IVW | 0.990 (0.931-1.054) | 0.763 |
|  | 7 | MR-Egger | 1.053 (0.626-1.771) | 0.853 |
|  | 7 | Weighted median | 0.982 (0.904-1.066) | 0.661 |
|  | 7 | Simple mode | 0.949 (0.828-1.089) | 0.487 |

CI, confidence interval; IVW, inverse variance weighted; MR-Egger, Mendelian randomization Egger; OR, odds ratio.
